# Difficulty Removing a Urethral Catheter in a Boy Due to a “Hairpin-Fold” Deformity: A Case Report

**DOI:** 10.7759/cureus.93216

**Published:** 2025-09-25

**Authors:** Akiko Kawano, Eriko Okunuki, Shuntaro Oka, Jun Hoshi, Satoshi Masutani

**Affiliations:** 1 Pediatrics, Medical Facility for Children with Profound Intellectual and Multiple Disabilities Karugamo no Ie, Fukushikai, Saitama Medical University, Saitama, JPN; 2 Pediatrics, Saitama Medical Center, Saitama Medical University, Saitama, JPN; 3 Nursing, Medical Facility for Children with Profound Intellectual and Multiple Disabilities Karugamo no Ie, Fukushikai, Saitama Medical University, Saitama, JPN

**Keywords:** complication, difficult removal, “hairpin-fold” shape, urethral catheter, urethral catheterization

## Abstract

Urethral catheterization is a common procedure performed in pediatric patients. We encountered a 21-month-old boy who had difficulty removing a urethral catheter because of the formation of a bent shape during catheter removal. The circumstances and ultrasonographic findings indicated that the deeply inserted catheter had formed a *hairpin-folded* shape inside the bladder and was being pulled out. The tip of the folded catheter was inserted into the curved part near the symphysis pubis, which was assumed to make the catheter difficult to remove. The appropriate insertion length of the urethral catheter depends on the patient’s age, sex, and whether a balloon is present or not. Therefore, we should be aware that such difficulty in removal can occur when a catheter is inserted deeper than necessary. The appropriate insertion length for each child must be considered when inserting a urethral catheter.

## Introduction

A urethral catheter is inserted into the bladder via the urethra to guide urine from the body. There are two types of urethral catheterization: clean intermittent catheterization (CIC) and indwelling urethral catheterization. Indications for CIC include aseptic urine collection, cystourethrography, neurogenic bladder, and relief of urinary retention. Indwelling urethral catheterization is used for the management of critically ill patients.

In particular, CIC is medically necessary for obtaining urine samples from young children who cannot urinate on command. Therefore, CIC is commonly performed in pediatric patients. Catheters come in a variety of shapes and sizes, depending on their intended purpose. Nelaton catheters are used for transient catheterization, whereas Foley catheters with balloon structures are used in indwelling urethral catheterization.

The bladder is pyramid-shaped and mildly distended. The base of the bladder, which is pyramid-shaped, faces the rectum in males and the vagina in females. The bladder base forms a triangle with its apex pointing downward. The ureters enter either side of the base (ureteral orifices), whereas the urethra emerges from the apex (internal urethral orifice). The narrowed portion of the urethra is referred to as the bladder neck. The location and anatomy of the urethra vary widely according to sex. In girls, the urethral opening is located below the clitoris, and the urethra is short and straight. After passing through the external sphincter, the urethra enters the bladder.

In boys, the urethral opening is located ventral to the penis, and the urethra is long. The male urethra is classified as the pendulous urethra, the bulbous urethra, the membranous urethra, and the prostatic urethra from the distal side. The urethra has an S-shaped curve behind the symphysis pubis. After passing through the external sphincter, the urethra enters the bladder. When the catheter is inserted into the urethra, resistance is felt near the base of the penis, and further resistance is felt with the contraction of the external bladder sphincter. The external urethral sphincter is a voluntary muscle that may contract when a patient strains during catheter insertion, making it difficult for the catheter to pass. Catheter insertion is relatively easy in girls, provided the location of the urethral opening is confirmed. However, insertion is more challenging in boys than in girls because the urethra is longer and more curved.

Complications of urethral catheterization include urethral injury, urinary tract infection, and difficulty in removing urethral catheters [1]. The incidence of urethral injury associated with catheter insertion has been reported to be 1.34% in adults [2], although this has not been observed in children. Urethral injury occurs when the catheter used is too large for the urethral diameter or when the cuff of the balloon catheter is inflated in the urethra. Invasive procedures, such as cystostomy or urethroplasty, are required if severe urethral injury occurs. The causes of difficult catheter removal include stone adhesion to the catheter and the inability to remove water from the balloon. Spontaneous knotting of a catheter, due to excessive insertion into the bladder, has been reported as a rare cause of removal difficulty [3-7].

Regarding the mechanism of knotting, it is believed that a deeply inserted catheter becomes overwound when it contacts the bladder wall, the distal tip of the catheter passes through an open loop, and the coil tightens into a knot as the catheter is withdrawn [6]. Knotting has been reported not only with straight catheters, but also with Foley catheters [3]. Although catheter knotting has been reported in adults, most reported cases, though rare, have occurred in neonates and children, particularly boys, whose urethral length is more likely to be overestimated [3]. However, to our knowledge, difficulty in removing urethral catheters owing to the formation of a *hairpin-fold* shape in the bladder has not been reported. Similar to catheter knotting, this case involving a *hairpin-fold* shape catheter is also thought to have resulted from excessive insertion into the bladder, which caused the catheter to bend back in the bladder. Here, we report a case of difficulty in removing a urethral catheter caused by the formation of a *hairpin-fold* shape.

## Case presentation

A 21-month-old boy (height 88 cm, weight 16 kg) with severe neonatal asphyxia, who had undergone simple tracheostomy and ventilatory management along with central diabetes insipidus, underwent clean intermittent catheterization twice daily by nurses. This was performed to reduce the risk of urinary tract infection caused by residual urine in addition to timely bladder expression. He was unable to move voluntarily and required assistance with all daily activities in addition to medical care.

**Figure 1:**
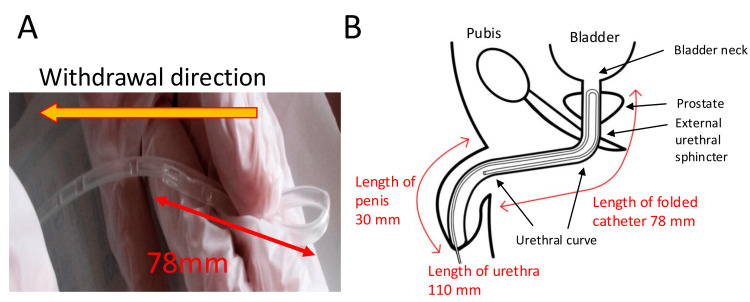
Urinary catheter with a hairpin curve and corresponding anatomic structure. (A) Catheter immediately after removal. (B) Assumed position of the catheter during difficulty in removal. Image credits: Akiko Kawano and Satoshi Masutani.

 An 8Fr urethral catheter (Nipro, Nelaton Catheter S®, Osaka, Japan) was inserted without resistance by a nurse, and urine with normal appearance was drained. Although the nurse initially pulled the catheter smoothly, she experienced resistance and difficulty in removing it, prompting a call from a pediatrician. The pediatrician attempted to remove the catheter; however, it could not be withdrawn. Ultrasonography (Toshiba, Japan; Aplio 400, convex probe) revealed that the catheter was not in the bladder but in the urethra just below it (image not recorded). On ultrasound, the catheter appeared to be in a single straight line, and no hairpin bending of the catheter was observed. Because the pediatrician confirmed that the catheter had already been withdrawn from the bladder, knotting was considered unlikely. The pediatrician then carefully withdrew the catheter, gently manipulating it by pushing and pulling without applying excessive force. The resistance decreased, and the catheter was eventually removed. Upon removal, the catheter was found to be folded, forming a ‘hairpin-fold’ shape 78 mm from the tip, with a distinct fold (Figure 1A). No complications, such as urethral injury or hematuria, were observed.

When the catheter was reinserted the next day, the urethral length was checked by ultrasound monitoring and was found to be 110 mm. Considering that the folded length was 78 mm and the penile length was 30 mm, the bending point of the catheter (the U-shaped portion) was located within the urethra, just below the bladder, consistent with the ultrasonographic findings at the time of difficult removal. The tip of the folded catheter may have been positioned approximately 30 mm from the external urethral opening near the base of the penis, where the urethra curves into an S-shape behind the symphysis pubis (Figure 1B). The tip of the folded catheter probably became caught on the curved portion of the urethra, causing resistance and preventing further catheter removal.

## Discussion

In the present case, removal of a folded, hairpin-shaped urethral catheter was difficult. To the best of our knowledge, this is the first reported case of a folded, hairpin-shaped urethral catheter that was difficult to remove. Since identifying the precise mechanism based on this case alone is challenging, we refer to reports on catheter knotting, which is believed to involve a similar mechanism. Regarding proposed mechanisms of catheter knotting, it is generally believed that the catheter tip passes through an open loop formed by over-winding when the catheter contacts the bladder wall. Upon withdrawal, this coil tightens into a knot [4]. Raveenthiran conducted simulation experiments using a balloon model to elucidate the mechanism of the knotting of a catheter. The author observed that the tip of the catheter swirled under the influence of water flow when the water was drained, and the tip approached a near-knotting position. Reported risk factors for knotting include the use of a catheter smaller than 10Fr, bladder overdistension, and insertion of a catheter longer than 100 mm into the bladder. The author further stated that urine flow plays an important role in knot formation [6].

**Figure 2:**
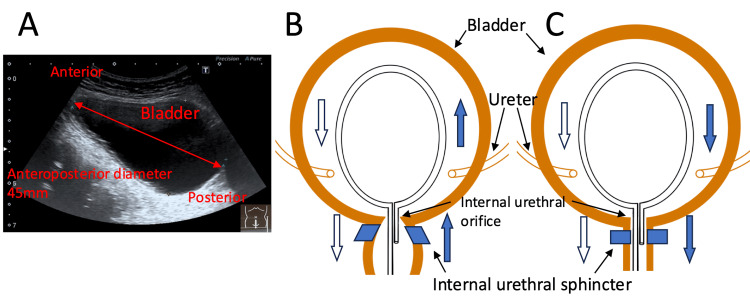
Bladder and possible mechanisms of hairpin-shaped formation. (A) Ultrasonography image of the bladder after the episode. (B) A deeply inserted catheter swirled in the bladder, with its tip accidentally lodged in the internal urethral orifice during withdrawal. (C) Contraction of the internal urethral sphincter prevents the catheter tip from returning to the bladder, directing it toward the external urethral opening and forming a hairpin shape. Image credits: Akiko Kawano and Satoshi Masutani.

We speculate that, in our case, the deeply inserted catheter was looped as it abutted the bladder wall, and its tip was directed toward the bladder neck. The tip of the catheter may have been swirled by the flow of water, and instead of passing through the open loop, it may have inadvertently entered the internal urethral orifice (Figure 2B).

Subsequently, it was hypothesized that (1) the involuntary muscles of the internal urethral sphincter contracted strongly during catheter removal (Figure 2C); (2) the tip of the catheter moved toward the urethra instead of the bladder owing to increased urethral friction; and (3) the folded shape formed during removal (Figure 1B).

It was presumed that the catheter passed through the urethra in a hairpin configuration and halted at the S-shaped curve behind the symphysis pubis. This region is known to experience resistance during catheter insertion [8]. Based on these assumptions and measurements, we estimated that the catheter insertion length from the external urethral opening at the time of the event was approximately 270 mm, and the minimum length of the catheter inside the bladder was approximately 160 mm. Since it is impossible to estimate bladder capacity immediately before the onset of complications, we performed bladder ultrasonography at a later date, before CIC, revealing that the anteroposterior diameter of the sagittal section of the bladder was 45 mm (Figure 2A). The minimum length of the catheter inside (160 mm) was approximately 3.5 times the anteroposterior diameter of the bladder. We speculate that the diameter of the bladder (45 mm) indicates that the catheter (160 mm) inserted into the bladder cannot remain straight and is forced to bend, causing its tip to point toward the bladder neck. The ultrasonographic findings support these hypotheses.

Our case had three risk factors for catheter knotting: catheter size, deep catheter insertion into the bladder, and sex. It is difficult to determine by this case alone whether the risk factors for the formation of hairpin-folded shapes are identical to those for knotting. However, this complication is unlikely to occur in the absence of deep catheter insertion.

The estimated dimensions were based on the following assumptions: the hairpin-folded U-shaped portion was located just below the bladder, the urethral length was 110 mm, the length of the hairpin fold was 78 mm, and the penile length was 30 mm. It is highly likely that our urethral length measurement was inaccurate. Although two physicians carefully measured the urethral length using ultrasonography, minor measurement inaccuracies could not be excluded entirely. Several formulas exist for estimating urethral length, such as: urethral length = 8.7 + age (in years) × 0.55 [9]. Based on this formula, the estimated urethral length in this case was 9.7 cm, which was not significantly different from our measurements. If the urethral length was shorter than 110 mm, the tip of the hairpin would be located at the pendulous urethra of the penile portion; if the urethral length was longer than 110 mm, it would be located on the bladder side above the penile base. In both cases, the tip of the hairpin was expected to be located in a relatively straight urethra; however, we hypothesized that if the tip of the hairpin at the urethra was turned outward, it might become caught on the urethra and stop moving forward.

In our case, the catheter was not intentionally inserted deeply. However, a thin pediatric urethral catheter may exhibit a time lag between insertion into the bladder and confirmation of urine drainage. This delay can lead to deeper-than-necessary insertion. In this case, the nurse did not advance the catheter further after noticing the urine backflow during catheter insertion.

Standards for urinary catheter insertion length are not well-established because they depend on the presence or absence of a balloon and the patient’s age, body size, and sex. Although shallow insertion of balloon catheters can cause urethral injury, deeper insertion is unnecessary for temporary non-balloon catheters. The incidence of spontaneous catheter knotting was 0.2 per 100,000 catheterizations [5]. Difficulty in removing a catheter due to a “hairpin-shaped” fold may be even rarer than the occurrence of catheter knotting. Although these complications are rare, awareness of the risks associated with deep insertion is crucial.

If catheter-removal difficulties are encountered during CIC, the possibility of a knotted or hairpin-folded catheter should be considered (Table 1). If a catheter nodule or knotted catheter is visible in the bladder during removal, a consultation with a urologist is required. If the catheter is not visible in the bladder on ultrasound, a hairpin-folded catheter, as in this case, should be considered a possible cause of difficulty in removal. However, one instance has been documented in which a knotted catheter was moved into the urethra, making its removal during passage through the urethra difficult [7]. Hence, catheter knotting cannot be ruled out because the catheter is not visible in the bladder or because initial removal was smooth [7]. However, in our case, the catheter was initially withdrawn smoothly, forming a hairpin-folded shape, before obstruction was encountered. The first step is to advance the catheter back into the bladder under ultrasound guidance. If the attempt is unsuccessful, the obstruction may be relieved by altering the angle of the penis or by gently rotating the catheter. Strong traction may cause urethral injury when knotting occurs in the urethra. It is essential to proceed with further steps to clarify catheter configuration through imaging studies, such as X-rays or CT scans, if the above methods fail to remove the catheter.

**Table 1:** Comparison of characteristics between knotted and hairpin-folded catheters.

	Knotting catheter	Hairpin-folded shape catheter
Frequency	・0.2 per 100,000	・Rare (this case only)
Risk factor	・Catheters smaller than 10Fr ・Overdistended bladder ・Excessive length insertion ・Male ・Neonates and children	・Indefinite
Findings of bladder ultrasonography	・Typically, a catheter can be found in the bladder. ・In rare cases, the catheter may not be identifiable within the bladder after migration into the urethra, even when it has a knotted configuration.	・Unable to identify the catheter in the bladder
Treatment	・Traction under anesthesia ・Unraveling the knot using a guidewire ・Endoscopic retrieval ・Suprapubic cystotomy	・Gentle traction with manipulation

In this case, repositioning the catheter into the bladder was difficult. The catheter was eventually removed without causing urinary tract injury. Regarding the reasons for successful removal, we hypothesized that the urethra was brought closer to a straight line owing to the repositioning of the penis, and the urethral sphincter was relaxed.

This study had some limitations. Bladder capacity was not measured using ultrasound during this event, although an overdistended bladder is considered a risk factor for knotting. The exact cause remains unclear, and further accumulation of cases is essential for future research.

## Conclusions

Here, we present a case of difficulty removing a urinary catheter due to its folding into a *hairpin-shaped *structure. If ultrasonography cannot explain the well-known causes of difficulty in removal, such as knotting of the catheter, a *hairpin-shaped* fold should be considered in the differential diagnosis. Ultrasound monitoring may play an important role in assessing the cause of difficulty in removal. In this case, changing the penile angle facilitated catheter removal. However, further validation is required to determine the effectiveness of this approach. To further understand the mechanism by which urethral catheters bend to form a *hairpin shape* and establish preventive procedures and removal methods, it is essential to accumulate similar cases in the future.
